# The Target Selective Neural Response — Similarity, Ambiguity, and Learning Effects

**DOI:** 10.1371/journal.pone.0002520

**Published:** 2008-06-25

**Authors:** Adam Hampshire, Russell Thompson, John Duncan, Adrian M. Owen

**Affiliations:** MRC Cognition & Brain Sciences Unit, Cambridge, Cambridgeshire, United Kingdom; University of Granada, Spain

## Abstract

A network of frontal and parietal brain regions is commonly recruited during tasks that require the deliberate ‘top-down’ control of thought and action. Previously, using simple target detection, we have demonstrated that within this frontoparietal network, the right ventrolateral prefrontal cortex (VLPFC) in particular is sensitive to the presentation of target objects. Here, we use a range of target/non-target morphs to plot the target selective response within distinct frontoparietal sub-regions in greater detail. The increased resolution allows us to examine the extent to which different cognitive factors can predict the blood oxygenation level dependent (BOLD) response to targets. Our results reveal that both probability of positive identification (similarity to target) and proximity to the 50% decision boundary (ambiguity) are significant predictors of BOLD signal change, particularly in the right VLPFC. Furthermore, the profile of target related signal change is not static, with the degree of selectivity increasing as the task becomes familiar. These findings demonstrate that frontoparietal sub-regions are recruited under increased cognitive demand and that when recruited, they adapt, using both fast and slow mechanisms, to selectively respond to those items that are of the most relevance to current intentions.

## Introduction

It is now widely accepted that a network of brain regions, distributed across the frontal and parietal cortices, form the components of an adaptable global system for the deliberate and intentional control of thought and action. This global ‘executive’ system underlies the flexibility of human behaviour, by enabling us to deliberately and selectively focus our attention on those items that are currently of relevance to the task at hand [1], [2], [3]. Whilst the existence of this network is no longer controversial, the contributions made by the anatomically distinct components from which it is comprised remain poorly defined. For example, to date, there have been several influential models proposing a dorsal-ventral axis across the lateral portion of the prefrontal cortex. These include the suggestion that the dorsolateral prefrontal cortex (DLPFC) and the ventrolateral prefrontal cortex (VLPFC) are differentially involved in exogenous vs. endogenous attentional orienting [4], first order vs. higher order executive functions [5], [6], [7], and the active maintenance vs. the controlled manipulation of items in working memory [8], [9]. Much of the current confusion regarding the precise nature of frontoparietal organisation results from the use of complex and cognitively heterogeneous task manipulations when attempting to functionally dissociate frontoparietal sub-regions. Hence, functional dissociations are often hard to interpret, with the (sometimes rather specific) cognitive functions that the tasks seek to examine typically being confounded with other more global parameters such as the general level of difficulty, and the overall level of engagement [10].

Target detection paradigms, in which the individual monitors a sequence of distractor objects for a learnt target stimulus, allow the effects of the relevance of the attended stimulus to the current task set to be examined whilst minimising variations in the complexity of required task parameters from one condition to another. Previously, we have examined the way in which different sub-regions of the frontoparietal network tune to respond selectively to the presentation of a frequently redefined target object whilst undertaking a simple event-related fMRI task [11]. We reported that whilst regions across the frontoparietal network were sensitive to the presentation of current target objects, their response was not homogeneous. The VLPFC, particularly in the right hemisphere, responded with a high degree of specificity to the current target object.

**Figure 1:**
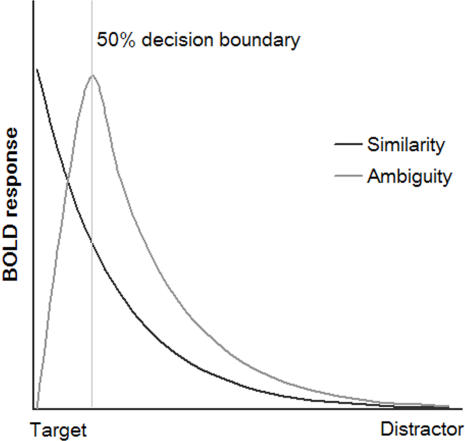
BOLD response functions as predicted by the working memory and difficulty hypotheses. Figure 1 illustrates hypothesised BOLD response functions. The BOLD response in sub-regions of the frontoparietal network could be predicted according to one of two distinct hypotheses. If a frontoparietal brain region tunes to represent the current task set, then a BOLD response function relating to the probability of positive identification should be observed (similarity to target; black). Alternatively, if a frontoparietal brain region is recruited during increased cognitive demand, then a function related to distance from the 50% target/distractor decision boundary, where the target-distractor discrimination is at its most ambiguous should be observed (ambiguity; grey). To mimic conditions in the current experiment (see later), here the decision boundary is drawn close to the target.

Whilst our previous results clearly identify the VLPFC as being particularly sensitive to the presentation of current targets, a number of questions regarding the precise nature of that sensitivity remain unresolved. Most importantly, in our previous task, the target selective response could be explained in terms of two popular hypotheses, which are commonly confounded. The first hypothesis - derived primarily from the findings of non-human primate single unit recording - relates to the type of information that is represented within the frontoparietal network. More specifically, it has been suggested that the frontoparietal network rapidly adapts to code for those items that are relevant to the currently intended goal [12], [13], [14], [15], [16]. In this case it would be predicted that the target selective response should resemble a simple similarity function, with the level of response related directly to the level of congruence between the currently attended object, and the recently defined target. An alternative hypothesis, however, derived primarily from neuroimaging research, refers to the type of cognitive demands under which the frontoparietal network is typically recruited. It has been observed that the BOLD signal within this network increases when the level of difficulty is parametrically varied across a wide variety of different task contexts [17]. On this basis, it has been proposed that the frontoparietal network forms a global system for attention that is engaged whenever the general level of difficulty increases and effortful executive control is exercised [1], [17], [18]. If the difficulty hypothesis is correct, then the BOLD response of frontoparietal regions should be predicted by the proximity of the currently presented object to the 50% target/distractor decision boundary, where the response decision is at its most ambiguous (figure 1).

Another pertinent question relates to the fact that much of our current understanding of the nature of the information represented within the lateral prefrontal cortex comes from the non-human primate electrophysiology literature. The tasks used in these studies are almost invariably extensively pre-trained to ensure good task performance. Herein lies a question regarding the relevance of results from these studies when seeking to understand how the frontoparietal network contributes to normal human behaviour. The frontoparietal cortex has often been proposed to play a particularly important role in *novel situations* by exerting deliberate ‘top-down’ or ‘executive’ control over those systems that would otherwise be governed by more habitual/learnt responses [19], [20], [21]. This top-down executive influence from the frontoparietal network thereby facilitates flexible/adaptable behaviour. A further question, therefore, concerns whether the target selective response within frontoparietal sub-regions varies as a function of increasing task familiarity, and if so, in what way?

Here, we addressed these questions using a modified version of our original task design. Volunteers monitored sequences of visually displayed objects for the presentation of a current target item. Distractor stimuli were morphed at varying degrees of similarity to the current target object, and the BOLD response could therefore be measured at each of these degrees of similarity. As the 50% decision boundary and the target object were at different positions on this similarity scale, it was possible to examine whether functions corresponding to the probability of positive identification (similarity) and distance from the 50% decision boundary (ambiguity) played significant roles in predicting the BOLD response. Furthermore, because volunteers undertook three identical blocks of experimental acquisition, it was possible to examine how the selective tuning functions varied as the task became increasingly familiar.

## Results

### Behavioural results

**Figure 2:**
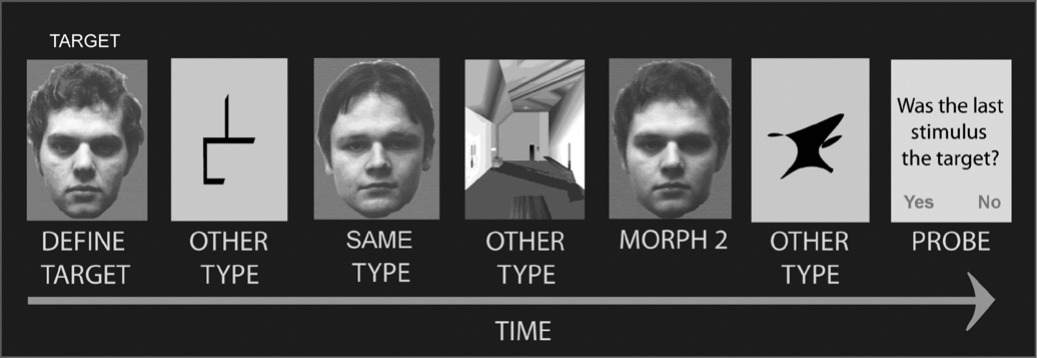
Task design. Volunteers passively monitored variable length sequences of objects for a current target item. Overt responses were made only when probed at the end of the sequence, subsequent to which a new target object was defined. The monitored stimuli could be at one of six degrees of similarity to the target, these being; the target object, a morph at one of three distances between the target and another object of the same category, a distractor from the target category (“same type”), or a distractor from a different category to the target (“other type”).

**Figure 3:**
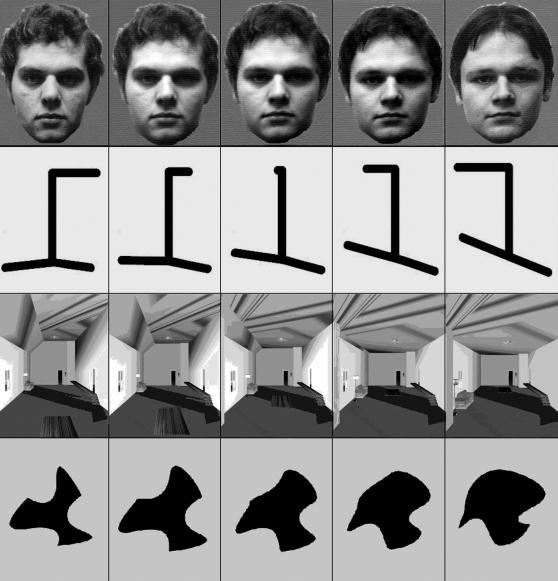
Stimulus set examples. Examples of one set of morphs from each of the four stimulus categories. The entire stimulus set comprised five standard objects from each of face, room, line figure, and abstract shape categories, as well as morphs formed at 3 degrees of similarity between all standard objects from within a given category. Running from left to right columns in the figure are a standard object, morphs 1 to 3, and a second standard object.

Twenty volunteers monitored sequences of visually displayed objects for the presentation of a current target item (figure 2). At the beginning of each sequence a new target item was presented with the word ‘target’, subsequent to which presentation of objects began. Responses, however, were made only when cued at the end of the sequence. Responses were cued by the question ‘was the last stimulus the target?’ and referred only to the last object. In this way, all critical events were kept free from overt motor activity. The lengths of the sequences were varied unpredictably, and within a given sequence, the current target could appear at any or multiple points to ensure attention throughout. To allow the target selective BOLD response to be examined in detail, monitored sequences were comprised of objects at six degrees of similarity to the current target, these being; the current target object (target), morphs one through three, distractors from the same category as the target (same type), and distractors from a different category to the target (other type) (figure 3).

**Figure 4:**
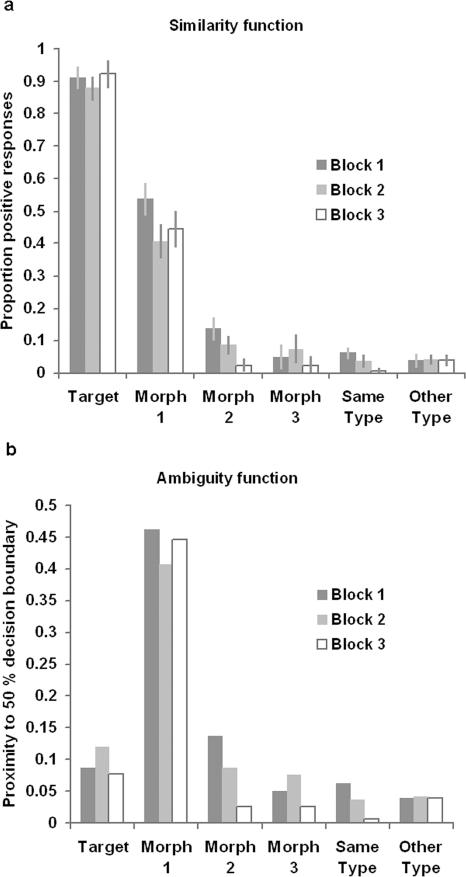
Behavioural results and predictor functions. Figure 4 illustrates the two cognitive predictor functions. 4a) The ‘similarity’ function at the top represents the probability of positive vs. negative response at probe for the six degrees of similarity to the current target item. Error bars display the standard error of the mean. 4b) The function at the bottom is a transform of the similarity function, and represents ‘ambiguity’ i.e. the distance from a 50% probability of being identified as the current target object.

In the behavioural analysis, the proportions of positive responses were examined in an ANOVA in which the conditions were similarity (target, morph 1, morph 2, morph 3, same type, other type)*experimental acquisition block (blocks 1 through 3) (Figure 4a). The analysis showed a significant interaction of block*similarity (F_(1,19)_ = 4.98 p<0.05), a significant main effect of similarity (F_(1,19)_ = 525.41 p<0.001), and a main effect of block (F_(1,19)_ = 16.11 p<0.001). The pair-wise comparisons between block 1 and 3 confirmed this result with significantly lower probabilities of positive response for morph 2 and same type distractors in the final block (target t = 0.25 p = 0.80; morph 1 t = −1.52. p = 0.14; morph 2 t = −2.90. p<0.01; morph 3 t = −1.00 p = 0.32; same type t = −3.94 p<0.001; other type t = 0.04 p = 0.97). In general the behavioural data reveal a small but significant trend towards increased selectivity across the three blocks.

### Plotting the target selective tuning functions in sub-regions of the frontoparietal network

**Figure 5:**
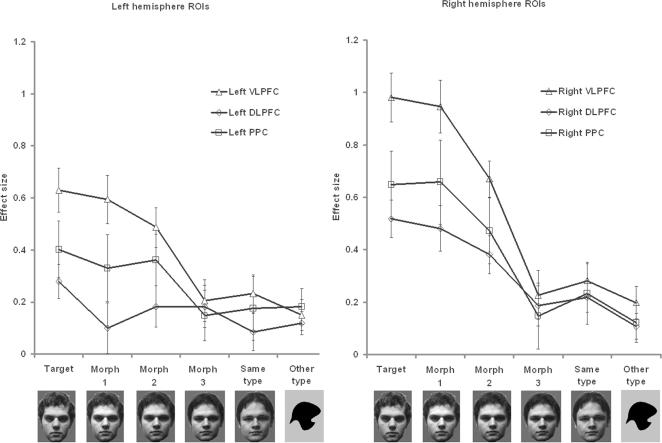
Plotting the target selective response in frontoparietal sub-regions. Figure 5 illustrates the selective tuning functions within the frontoparietal ROIs averaged across the three blocks of scanning acquisition. The right VLPFC followed the steepest tuning function and there was a lateralisation effect favouring greater response to targets in the right hemisphere ROIs. Error bars display the standard error of the mean.

Our first analysis examined responses to the different possible stimulus types in order to examine the question of whether target selective tuning functions varied between different frontoparietal sub-regions, and also to examine whether they varied within those sub-regions across the three blocks of experimental acquisition. Group level analyses were carried out using focused regions of interest (ROIs) representing the DLPFC, the VLPFC, and the posterior parietal cortex (PPC). Data extracted from the frontal and parietal ROIs for the presentation of targets, morphs, and distractors, were examined using repeated measures analyses of variance (ANOVA). The first ANOVA examined the effects of similarity to the current target object averaged over the three blocks of experimental acquisition (Figure 5). The conditions were ROI (VLPFC, DLPFC, PPC)*hemisphere (left, right)*similarity to the target (target, morph 1, morph 2, morph 3, same type, other type). The within subject effects revealed a significant interaction of hemisphere*similarity (F_(1,19)_ = 17.34 p<0.001), and a significant interaction of ROI*similarity (F_(1,19)_ = 8.07 p<0.001). There were also significant main effects of similarity (F_(1,19)_ = 12.42 p<0.001), and hemisphere (F_(1,19)_ = 20.96 p<0.001), and ROI (F_(1,19)_ = 6.51 p<0.005). The interactions indicated that different ROIs followed different selective tuning functions, and they were therefore examined separately in a series of one way ANOVAs in which the condition was similarity (target, morph1, morph 2, morph 3, same type, other type). There were strong main effects of similarity in the ventrolateral prefrontal cortex, particularly in the right hemisphere (VLPFC left F_(1,19)_ = 12.31 p<0.001, VLPFC right F_(1,19)_ = 38.36 p<0.001). There were also significant main effects of similarity in the right DLPFC and the right PPC (DLPFC left F_(1,19)_ = 1.63 p = 0.16, DLPFC right F_(1,19)_ = 10.38 p<0.001; PPC left F_(1,19)_ = 1.98 p = 0.09, PPC right F_(1,19)_ = 10.79 p<0.001). Overall, the results revealed that within the frontoparietal network, the target selective response was greatest in the VLPFC with a general lateralisation effect favouring the right hemisphere.

### Examining the effects of task familiarity on the target selective tuning functions in the frontoparietal network

**Figure 6:**
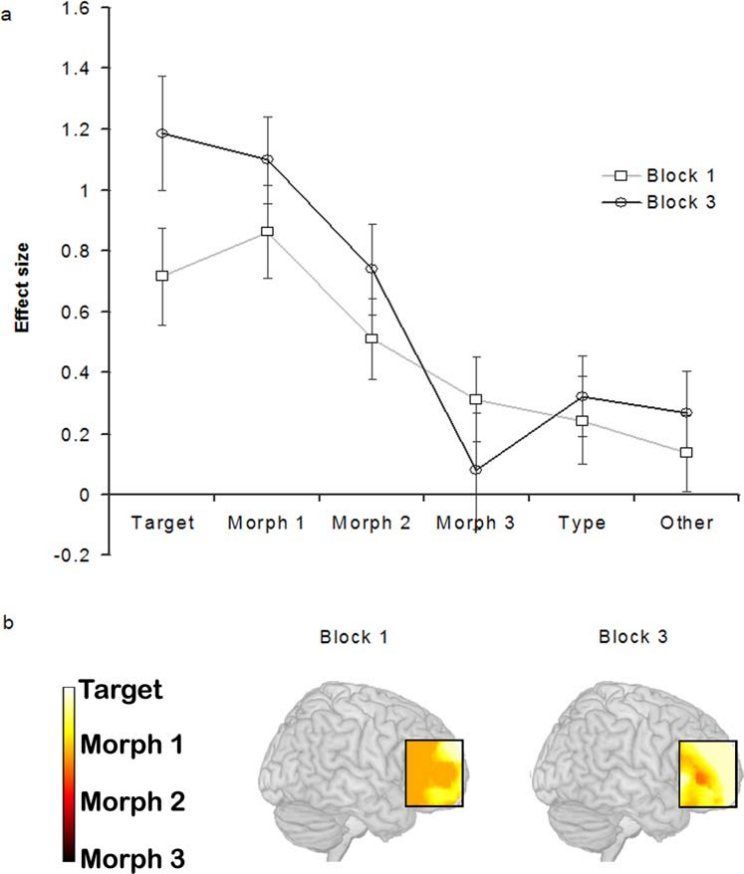
Learning effects in the right VLPFC. Figure 6a illustrates the tuning functions from the right VLPFC in the first acquisition block, when the task was novel, and the third acquisition block, where the task was most familiar. The peak of the tuning function shifted from morph 1 towards the target as the task and stimuli became more familiar. Error bars display the standard error of the mean. Figure 6b shows the average peak position of the tuning function in acquisition blocks 1 and 3 in the right lateral frontal cortex. The peak of the tuning function can be seen to shift from morph 1 in acquisition block 1 towards the target in acquisition block 3 across a large swathe of the right lateral frontal cortex.

The data were then examined for the effects of task familiarity. The ROIs were examined separately in a series of two way ANOVAs, in which the conditions were similarity (target, morph1, morph 2, morph 3, same type, other type)*experimental acquisition block (block 1, block 2, block 3). A significant interaction of similarity*acquisition block was observed in the right VLPFC (figure 6a) (left F_(1,19)_ = 2.22 p = 0.15; right F_(1,19)_ = 5.88 p<0.05). The other ROIs displayed no significant familiarity*similarity interactions. The nature of this learning effect would appear to be a shifting of the peak of the tuning function from morph 1 in the first acquisition block, towards the target in the third acquisition block (figure 6a). This shifting of the tuning function peak was explored further by calculating the average peak position on a voxel by voxel basis across the right lateral prefrontal cortex. A general shift in the peak position from morph 1 to the target was apparent across the lateral prefrontal cortex (figure 6b). This change in the peak position of the tuning function could be accounted for by a general shifting in frontoparietal resources away from resolving the ambiguous target-distractor decision at morph 1 and towards recognition of the target as the task becomes more familiar.

For block 1, a direct contrast between morph 1 distractors and the target generated no significant results. To investigate whether this effect was more reliable when the task was at its most novel, the data were remodelled for the first half of session 1 only. Contrasting morph 1 distractors vs. the target using FDR correction for the whole brain mass at p = 0.05 revealed a significantly greater BOLD response in the right IFG (x = 46 y = 10 z = 26 and x = 34 y = 26 z = −4), in the left IFG (x = −36 y = 18 z = −2), and in the right PPC (x = 30 y = −58 z = −56). This finding confirms that the resolution of ambiguous target-distractor decisions recruited frontoparietal resources to a particularly large extent when the task was novel.

### Ambiguity and similarity as predictor functions

A further analysis was carried out to test whether the BOLD response in frontal and parietal sub-regions was best accounted for in terms of a) perceptual similarity to the current target object, b) the difficulty of the current target/distractor discrimination, or c) a combination of these two cognitive factors. To address this issue, we examined the extent to which functions derived from the behavioural data (see methods) representing the probability of positive identification (similarity to the target - figure 3a), and proximity to the 50% decision boundary (degree of ambiguity – figure 3b), could predict the BOLD response within the same frontoparietal sub-regions. Group level analyses were carried out using the focused ROIs representing the DLPFC, the VLPFC, and the PPC. In each case, regressors were formed by weighting the onsets and durations of stimulus presentation with the behavioural similarity and ambiguity functions prior to convolution with the canonical haemodynamic response function (see methods).

**Figure 7:**
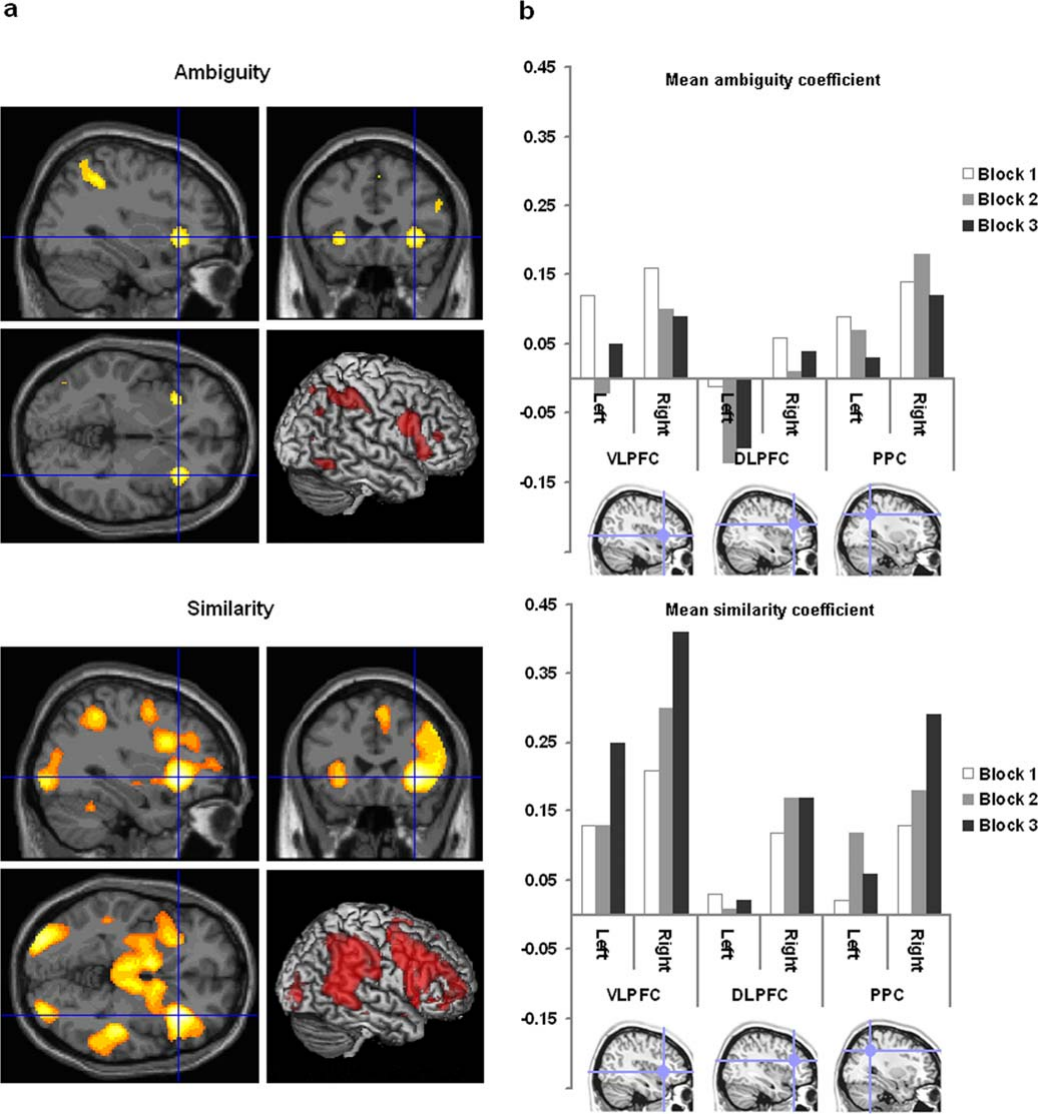
Significant effects of the ambiguity and similarity predictor functions. Figure 7 illustrates brain regions in which the BOLD response is significantly predicted by the ambiguity regressor (top), and the similarity regressor (bottom). 7a) On the left side are results from the unconstrained whole brain analysis, collapsed across the three blocks of experimental acquisition with FDR correction at p = 0.05 for the whole brain mass. 7b) On the right side are the results from the focused ROI analysis, calculated separately for each acquisition block. The relative weightings in the frontoparietal ROIs shifted away from ambiguity and towards similarity as a function of task familiarity. Error bars display the standard error of the mean.

In the group level analysis, we first examined each frontoparietal ROI for the positive effects of ambiguity averaged across the three acquisition blocks. Ambiguity played a significant role in predicting the BOLD response in both the right VLPFC, and right PPC (right VLPFC t = 2.45 p<0.01; right PPC t = 2.65 p<0.005). Whole brain analysis (FDR corrected for the whole brain mass at p = 0.05) confirmed the results from the ROI analysis, with significant BOLD activation in the ventrolateral prefrontal cortex bilaterally (Figure 7). It should be noted that the peak VLPFC co-ordinates for the ambiguity regressor were located posterior and medial to our ROI between BA 44/BA 47 and the anterior insula (table 1), and the activation spread across the anterior insula and the inferior operculum. There were also significant activation peaks in the right DLPFC, right pre-motor cortex, right PPC and right occipital cortex.

**Table 1:** Peak co-ordinates from the whole brain analysis.

Ambiguity						
X	y	z	t	p (FDR)	Anatomical region	Approximate BA
−30	22	−4	4.24	0.007	Inferior Frontal Gyrus	BA 47
34	24	−2	4.66	0.002	Inferior Frontal Gyrus	BA 47
48	38	14	3.59	0.028	Inferior Frontal Gyrus	BA 46
50	10	26	5.36	p<0.001	Inferior Frontal Gyrus	BA 44
44	−38	50	4.41	0.004	Inferior Parietal Lobule	BA 40
50	−62	−10	4.02	0.011	Middle Occipital Gyrus	BA 19
Similarity						
x	y	z	t	p (FDR)	Anatomical region	Approximate BA
34	24	−2	7.01	p<0.001	Inferior Frontal Gyrus	BA 47
40	16	26	6.42	p<0.001	Middle Frontal Gyrus	BA 46
46	4	48	6.19	p<0.001	Middle Frontal Gyrus	BA 6
−6	−2	58	5.31	p<0.001	Medial Frontal Gyrus	BA 6
10	10	50	5.29	p<0.001	Medial Frontal Gyrus	BA 32
6	38	40	5.51	p<0.001	Medial Frontal Gyrus	BA 6
−30	18	−8	5.1	p<0.001	Extra-Nuclear	BA 13
−50	−44	28	5.51	p<0.001	Inferior Parietal Lobule	BA 40
66	−40	28	6.15	p<0.001	Inferior Parietal Lobule	BA 40
−26	−72	26	2.42	0.031	Precuneus	BA 31
−64	−20	34	4.62	p<0.001	Postcentral Gyrus	BA 1
−36	−24	50	5.13	p<0.001	Postcentral Gyrus	BA 3
−54	−50	12	5.36	p<0.001	Superior Temporal Gyrus	BA 22
48	−26	−6	6.6	p<0.001	Superior Temporal Gyrus	BA 21
−26	−94	−8	6.77	p<0.001	Inferior Occipital Gyrus	BA 18
30	−90	−6	6.02	p<0.001	Middle Occipital Gyrus	BA 18
−8	−74	8	2.53	0.025	Cuneus	BA 23
10	8	6	5.94	p<0.001	Caudate	
−20	−4	2	4.53	p<0.001	Lateral Globus Pallidus	
−10	2	0	6.13	p<0.001	Medial Globus Pallidus	
12	0	4	5.75	p<0.001	Lentiform Nucleus	
−6	−18	−4	5.44	p<0.001	Mid Brain	
8	−10	2	5.78	p<0.001	Thalamus	

(P values FDR corrected for the whole brain mass).

The frontoparietal ROIs were then examined for significant positive effects of similarity to the target averaged across the three acquisition blocks. There were large significant effects of similarity in the VLPFC bilaterally, the right DLPFC, and the right PPC (left VLPFC t = 4.01 p<0.001; right VLPFC t = 6.37 p<0.001; right DLPFC t = 3.52 p<0.001; right PPC t = 3.76 p<0.001). Whole brain analysis confirmed the results of the ROI analysis (Figure 7), with significant BOLD activation throughout much of the frontoparietal network for the positive effect of similarity, including the VLPFC bilaterally, the PPC bilaterally, and the right DLPFC. In addition, a network of other brain regions was activated, including visual cortex, temporal cortex, the anterior insula, pre-motor cortex, the anterior cingulate, the pre-SMA, and areas within the striatum (see table 1). Overall, therefore, the response within the frontoparietal network, particularly within the right VLPFC, was best predicted by a combination of both the ambiguity and the similarity functions, with similarity especially important.

Examination of the ROI data separately for each acquisition block indicated that there was a general trend towards increased weighting on the similarity regressor, and decreased weighting on the ambiguity regressor across the three acquisition blocks (Figure 7). We examined the significance of this trend in a full factorial model in SPM 5 in which the factors were predictor (ambiguity or similarity)*acquisition block (block 1, block 2, block 3). Our results revealed a significant interaction of acquisition block*predictor function in the VLPFC bilaterally (left F = 5.09, p<0.01; right F = 14.77, p<0.001), in the right DLPFC (left F = 0.92, p = 0.40; right F = 3.07, p = 0.05), and in the right PPC (left F = 2.31, p = 0.1; right F = 8.94, p<0.001), indicating that with practice, similarity becomes relatively more important than ambiguity in predicting the BOLD response across the frontoparietal network. The whole brain analysis did not reveal any peak activation foci for the block*predictor interaction at the corrected threshold.

## Discussion

The advantage of using a simple target detection paradigm to investigate frontoparietal function is that it enables the selectivity of the BOLD response to be examined whilst minimising differences in the complexity of the current task parameters. In this tightly controlled context, any observed results must be driven by the similarity of the currently attended stimulus to the object that is at the focus of currently intended behaviour (i.e. the target). Here, the use of target-distractor morphs has allowed us to examine the target selective BOLD response in the human frontoparietal network at a higher degree of acuity than has previously been possible. Our results reveal that a broad swathe of cortex rapidly adapts to respond selectively to the current target object. In line with models that posit a global/adaptive system for working memory and attention [1], [3], [18], [22] this ability appears to be generalised across different stimulus categories. The target selective network includes a large swathe of frontal and parietal cortex, including the PPC, the DLPFC, and the VLPFC. The selective tuning functions are not homogeneous throughout the frontoparietal network, however, with distinct sub-regions displaying significantly greater sensitivity to the current target object.

Previously, we have reported that the ventral portion of the lateral prefrontal cortex is particularly sensitive to the presentation of target objects [11]. On this basis, we have suggested a degree of specialisation within the frontoparietal network, with the more ventral and posterior portion of the lateral prefrontal cortex tuning to respond to those items that are at the current focus of intended action with a particularly high degree of selectivity. This specialisation is replicated here, with heightened activity in the VLPFC compared to other regions of the frontoparietal cortex, including the anatomically adjacent DLPFC. With the increased power afforded by the current design, however, it is clear that this apparent specialisation is quantitative as opposed to absolute, with other frontal and parietal regions following similar shaped tuning functions, but to a lesser extent.

Previously, we have also reported a lateralisation effect favouring the right hemisphere during target detection [11]. This finding is replicated again here, with heightened target related activation in the right hemisphere throughout the frontoparietal network. This lateralisation effect is most prominent in the DLPFC, with the left DLPFC appearing to be almost completely insensitive to the presentation of the current target object. Whilst it is now clear that frontal and parietal regions are consistently more activated in the right hemisphere during target detection, the question still remains whether this lateralisation effect is due to the right hemisphere being more involved in the detection of targets, or to the type of stimuli used. One way of testing the possibility that the lateralisation effect is due to the type of stimuli would be to replicate the current task design, but with words instead of objects. One might predict that, in such a situation, the lateralisation effect could be reversed to favour the left hemisphere. It is also important to note that the left DLPFC may play a less transient role in target detection, a hypothesis that cannot be tested here due to the rapid event related design not allowing the estimation of a resting state baseline.

In our previous study [11] the right DLPFC was observed to respond at a more categorical level than the VLPFC, with similar increases observed in the BOLD signal during the presentation of both targets and distractors from the same category. Here, the previous findings were only partially replicated, with the particular sensitivity of the right VLPFC to target objects appearing to be robust across experiments, but the wider tuning of the DLPFC appearing to be more sensitive to the exact task parameters. This lack of replication when task demands are changed is a running theme in studies that seek to functionally dissociate the DLPFC and the VLPFC. Hence, whilst dissociations have been reported [4], [8], [9], subsequent studies that use similar task manipulations often report that the VLPFC and DLPFC follow a similar activation profile [8], [23]. One relatively constant factor, however, is that when these anatomically distinct sub-regions of the lateral prefrontal cortex *are* functionally dissociated, the VLPFC tends to be implicated in simple executive functions, for example the maintenance of items in working memory [5], [6], [7], [8], [9], whereas the DLPFC tends to be implicated in more complex, although not necessarily more difficult task demands such as manipulating, monitoring, and structuring items in working memory [7], [8], [9], [24], [25]. It seems sensible to propose, therefore, that differences between the DLPFC and the VLPFC are statistical as opposed to absolute [1], with both brain regions capable of supporting similar cognitive processes. Under certain conditions, however, the roles played by these two brain regions may dissociate and when they do, they dissociate in a hierarchical manner.

This study was designed not only to replicate our previous findings, but also to address two key questions using the higher degree of acuity afforded by the use of morphed distractors. 1) Which cognitive factors can predict the target selective response in the frontoparietal network? 2) Are the target selective tuning functions static, or do they change as a function of task familiarity? We addressed the first of these questions using two cognitive predictor functions. The first, similarity, represented the probability of positive vs. negative response at each of the six degrees of similarity to the current target item. This similarity function relates most closely to findings from the electrophysiology literature in which frontal neurons have been observed to respond selectively to a broad range of task-relevant information, for example responses, rewards, and learnt target stimuli [3], [26], [27], [28], [29]. Based on the electrophysiology findings it seems sensible to predict that the better the currently attended object matches the item that the currently intended action plan is programmed around, the more it will activate the frontoparietal network, which is assumed to represent the currently relevant objects, actions, and task criteria. Another popular hypothesis posits that the frontoparietal network forms a highly adaptable system that is recruited whenever the general level of cognitive demand increases [1]. This latter hypothesis is repeatedly supported by the neuroimaging literature, which tends to reveal increased BOLD signal in the frontoparietal network when a wide variety of cognitive demands are parametrically increased [17]. The second cognitive predictor function was based, therefore, on how close the probability of target identification was to 50%: this was highest, when the target/distractor decision was at its most ambiguous, and resolution of this ambiguity required maximal processing. It is important to note that these two hypotheses are not mutually exclusive and it was our aim to disentangle them in order to test whether either or both played a significant role in predicting the BOLD response.

Our results demonstrate that both similarity to the target item and degree of ambiguity in the target/distractor decision play a significant role in predicting the target selective BOLD response. Our results did not simply show an activation profile sharply peaked for the target, as predicted by the similarity regressor (figure 1). Neither did they show a profile sharply peaked for the most ambiguous stimulus morph 1, as predicted by the ambiguity regressor (figure 1). Instead the balance of activity between target and morph 1 varied over regions and stages of practice.

It is clear from the results that the selective tuning functions are not static, particularly in the right VLPFC, where the BOLD response becomes increasingly selective as the task becomes more familiar. This increased selectivity can be interpreted in terms of a redistribution of cognitive resources. Hence, the ambiguity of the target-distractor decision places a particularly high demand on frontoparietal resources at the earliest stages of the task, when the task parameters and stimulus set are novel. Conversely, the similarity of the attended stimulus to the current target object plays a larger role in predicting the BOLD response in the later stages of the task, when the task parameters and stimulus set are familiar. Herein lies a question over the relevance of findings from much of the current electrophysiology literature when attempting to understand the contribution of the frontoparietal network to normal human behaviour. The results of selective frontal lobe lesions have often been used to suggest that the frontoparietal network plays a particularly important role when dealing with novel problems [19], [20]. However, the vast majority of electrophysiology experiments use extensive pre-training, and it seems sensible to suggest, therefore, that the findings from those studies relate to the way in which neurons within this network maintain attention to, and solve, routine, habitual problems. Our results would suggest that, with practice, frontoparietal processing related to ambiguity/cognitive demand is at best minimised, and may therefore appear to be less significant in heavily pre-trained studies. By contrast, the extent of adaptive tuning to the currently relevant objects increases with learning, and would therefore seem to be more representative of the frontoparietal role in attention in studies that employ over-learnt tasks. It remains to be answered whether the current effects of familiarity on the target selective response relate to learning of the stimulus set, or a lowering of general engagement as the task becomes increasingly familiar. In either case, our data confirm the importance of task familiarity in frontoparietal function.

An important aside is the relevance of the current findings to those studies of inhibitory control that have reported activation throughout a very similar network. Of particular relevance to the current findings is the increased BOLD response in the right VLPFC during the suppression of a routine motor output following an infrequent stop cue [30], [31]. During Go/NoGo tasks, the maintained task program is to look for an infrequent and previously learnt cue to stop, and on receiving that cue to interrupt a routine motor response. It is plausible to suggest that a large component of the ‘inhibition’ condition in the Go/NoGo task is recognition of the cue to stop, a process that is very similar to identifying a learnt target stimulus. The process of subsequently stopping the routine response is probably facilitated by the ‘top-down’ biasing signals that are widely held to be the primary mechanism by which control is exercised by the executive system [1], [32], [33], [34]. Whilst this process could be described as inhibition, it could also be described as the *implementation of the currently maintained task program*. In that respect, it should be noted that this manipulation differs from inhibitory control in the more classical sense of an *effortful change in the current task program,* which usually occurs as a consequence of previously rewarded responses leading to sub-optimal feedback from the environment. Inhibitory control in this more classic sense is known to rely on additional frontal lobe circuitry, most particularly sub-regions of the orbitofrontal cortices [35], [36], [37], [38].

Finally, we have presented here a working proof that tuning in simple target detection is a useful scale for measuring the degree of attentional focus in brain activity. Here this scale has been used to compare tuning functions across distinct frontoparietal regions of interest, and across varying levels of task familiarity. The same method may well be useful for testing a variety of hypotheses, for example, differences in attentional selectivity across clinical populations, and under varying cognitive and pharmacological conditions.

## Materials and Methods

### Experimental design

Volunteers were instructed to look for a visually displayed target object within sequences of distractor objects (figure 1). At the beginning of each sequence a new target item was presented with the word ‘target’ for 3400 ms. Subsequent to the target stimulus being defined, presentation of the sequence of targets and distractors began. Each item of the sequence was displayed for 1500 ms and was followed by an inter-stimulus-interval of 400 ms. Sequences were predefined and pseudo-randomised. The sequence length was varied unpredictably from 1 to 8 items, and within a given sequence, the current target could appear at any or multiple points. At the end of each sequence a probe stimulus consisting of the question ‘Was the last stimulus the target?’ was presented on the screen for 3400 ms, and volunteers were required to respond yes or no, using a button box with the first two digits of their right hand. The words ‘yes’ and ‘no’ also appeared below the probe, randomly assigned to the left and the right of the display, indicating which buttons to press for the positive and negative response. Critical contrasts were therefore kept free of overt motor activity, whilst attention was ensured throughout the monitored sequence. Twenty healthy right-handed volunteers between the ages of 20 and 40 undertook the fMRI task, which consisted of 3*12 minute blocks of scanning acquisition, each containing 40 stimulus sequences.

Targets, same category distractors, and other category distractors, were drawn from the same fixed set of stimuli, consisting of five standard objects from each of four distinct categories: faces, rooms, line figures, and abstract shapes (see figure 2). To allow the target selective BOLD response to be examined in detail, morphs were generated between all standard objects of the same category, at three physically equidistant degrees of similarity (for example stimuli see figure 2). The monitored sequence consisted, therefore, of objects at six degrees of similarity to the current target, these being; the current target object (target), morphs one through three, distractors from the same category as the target (same type), and distractors from a different category to the target (other type). Within each block of scanning acquisition, volunteers monitored 22 targets, 22 morphs from each of the three degrees of similarity, 22 same type distractors, and 74 other type distractors. Each standard object was used as the target twice in a block of scanning acquisition, and multiple times as a distractor. The presentation of targets, morphs, and distractors was balanced across the experimental block so that the relative probabilities were equivalent across all eight positions in the stimulus sequence, in this way averaging out any effects due to reconfiguration to a new target object, or the expectancy of an impending probe. The sequences were identical across the three experimental blocks to ensure maximum cross block comparability when examining the effects of learning.

### Scanning acquisition

Scanning was carried out at the MRC Cognition and Brain Sciences Unit using a 3 Tesla Siemens Tim Trio. 32*3 mm slices (1 mm inter-slice gap, descending slice order) were acquired in 2 seconds for each image (in-plane resolution 3×3 mm). 360 T2-weighted echo-planar images depicting BOLD contrast were acquired per block of scanning acquisition, with the first 10 discarded to avoid T1 equilibrium effects. The experiment was programmed in Visual Basic 6 and the display projected onto a screen, visible from the scanner via a mirror, with stimuli subtending a visual angle of approximately 6.5 degrees.

Images were pre-processed and analysed using the Statistical Parametric Mapping 5 software (SPM5, Wellcome Department of Cognitive Neurology). Prior to analysis, images were slice time corrected, reoriented to correct for subject motion, spatially normalised to the standard Montreal Neurological Institute template, smoothed with an 8 mm full-width at half-maximum Gaussian kernel, and high-pass filtered prior to analysis (cut-off period 180 s).

### Event modelling

Two separate fixed effects analyses were carried out on each volunteer's data using general linear models. The first design examined how the BOLD response varied when the participant was presented with objects at different degrees of similarity to the target. This model was used to examine the question of whether the selective tuning functions varied between the different frontoparietal sub-regions, and also to examine whether they varied within those sub-regions across the three blocks of experimental acquisition. 20 regressors were included in this model, with the onset and duration of each picture presentation event described according to three orthogonal parameters. For each event, the first descriptor was similarity to the target object, with 6 levels: target, a morph at one of the three degrees of similarity to the target, a ‘same type’ distractor, or an ‘other type’ distractor. The second descriptor was object category with 4 levels: faces, rooms, abstract line figures, and abstract shapes. The third descriptor was temporal position in the monitored sequence (positions 1 through 8). The target definition stage was included as a further regressor, and the final regressor was formed from the onsets and durations for the probes at the end of the sequences with the corresponding motor responses. Regressors were created by convolving these timing functions with a basis function representing the canonical haemodynamic response.

Group level analyses were carried out using focused regions of interest (ROIs) representing different sub-regions of the frontoparietal network. 10 mm radius spherical ROIs were defined bilaterally in the DLPFC, the VLPFC, and the PPC, based upon averaged coordinates taken from a previous analysis of common frontal and parietal activity associated with diverse cognitive demands [2], [17]. The centre points of these regions were located at ±38, 30, 22 for the DLPFC, ±39, 20, 2 for the VLPFC, and ±31, −51, 40 for the PPC. For each participant, the level of response to each of the six degrees of similarity to the target (targets, morph 1, morph 2, morph 3, same type, other type) was estimated using fixed effects analysis. These data were averaged across voxels within each of the ROIs using the MARSBAR toolbox [39], and the mean values were exported for analysis using SPSS.

The second linear model was identical to the first, except that the regressors corresponding to the six degrees of similarity to the target item were replaced with two new regressors, weighted according to two predictor functions, similarity and ambiguity. The behavioural data, averaged across all participants, from the responses to probes was used to plot the predictor functions, separately for each of the three acquisition blocks. The similarity function was defined by calculating the probability of a positive vs. a negative response across the six degrees of similarity to the target object. The ambiguity function was estimated from the response data by taking the un-signed result of 0.5 minus the probability of positive response, and then subtracting this value from 0.5. This renders a function that is maximal for a positive decision probability of 0.5, and at zero for a probability of 0 or 1. The onsets and durations for monitored objects were then weighted according to the two predictor functions, to form the two new regressors. These regressors were convolved with the canonical haemodynamic response function and, to control for scaling, were normalised by dividing by the root mean square value of the entire regressor before entry into the design matrix.

In the group level analysis, each volunteer contributed six whole brain images, containing the parameter estimates for the similarity and ambiguity regressors, separately for each block of scanning acquisition.
